# One year of active moss biomonitoring in the identification of PAHs in an urbanized area—prospects and implications

**DOI:** 10.1007/s11356-024-33831-8

**Published:** 2024-05-28

**Authors:** Paweł Świsłowski, Stanisław Wacławek, Vojtěch Antos, Inga Zinicovscaia, Małgorzata Rajfur, Maria Wacławek

**Affiliations:** 1 Institute of Biology, University of Opole, Kominka 6, 6a, 45-032, Opole, Poland; 2 Institute for Nanomaterials, Advanced Technologies and Innovation, Technical University of Liberec, Studentská 1402/2, 461 17, Liberec, Czech Republic; 3 Horia Hulubei National Institute for R&D in Physics and Nuclear Engineering, Bucharest Magurele, 30 Reactorului Str. MG-6, Bucharest, Romania; 4 The Institute of Chemistry, Moldova State University, 3 Academiei Str., 2028, Chisinau, Moldova; 5 Society of Ecological Chemistry and Engineering, Zawiszaków 3/103, 45-288, Opole, Poland

**Keywords:** Active biomonitoring, Air pollution, Biomonitor, Feathermoss, Polycyclic aromatic hydrocarbons

## Abstract

Classical monitoring of air pollution provides information on environmental quality but involves high costs. An alternative to this method is the use of bioindicators. The purpose of our work was to evaluate atmospheric aerosol pollution by selected polycyclic aromatic hydrocarbons conducted as part of annual active biomonitoring (“moss-bag” technique) with the use of three moss species: *Pleurozium schreberi*, *Sphagnum fallax*, and *Dicranum polysetum*. The gas chromatography-mass spectrometry (GC–MS) was utilized to determine certain 13 polycyclic aromatic hydrocarbons (PAHs). Three seasonal variations in PAH concentrations have been observed as a result of the study. A fire on the toilet paper plant caused an increase of five new compounds: benzo(k)fluoranthene (BkF), benzo(a)pyrene (BaP), indeno(1.2.3)-cd_pyrene (IP), dibenzo(a.h)anthracene (Dah), and benzo(g.h.i)perylene (Bghi) in proximity after 8 months of exposure compared to previous months. The effect of meteorological conditions on the deposition of PAHs (mainly wind direction) in mosses was confirmed by principal component analysis (PCA). *Dicranum polysetum* moss accumulated on average 26.5% more PAHs than the other species, which allows considering its broader use in active biomonitoring. The “moss-bag” technique demonstrates its feasibility in assessing the source of PAH air pollution in a long-term study. It is recommended to use this biological method as a valuable tool in air quality monitoring.

**Supplementary Information:**

The online version contains supplementary material available at 10.1007/s11356-024-33831-8.

## Introduction

The problem of air pollution with PAHs in Europe is well known (Schreiberová et al. 2020), and it entails negative consequences for people exposed to their harmful effects (Thangphatthanarungruang et al. 2023). Exposure of humans and animals to PAHs can lead to different effects depending on a diversity of coefficients (Sun et al. 2021). For fauna, PAH compounds are also toxic. Contents hazardous to, for example, benthic species, were conversely correlated with the hydrophobicity of single polycyclic aromatic hydrocarbons. These arrangements are essential for the environmental risk assessment of these compounds (Jesus et al. 2022). The presence of O, N, or S in PAH aromatic rings increased their toxicity also (Krzyszczak and Czech 2021). Concentrations of PAHs in the air depend mainly on their source and meteorological conditions (e.g., temperature). High levels of PAHs are mainly recorded in cities during winter—domestic heating (Bai et al. 2023). In addition, the most common sources of PAHs are the contribution of industrial gases and vehicle emissions, and natural sources are volcanic eruptions and forest fires (Lin et al. 2020). There is a real need to monitor the pollution of these compounds (Li et al. 2022), which is most often performed and evaluated from the perspective of long time periods (Albuquerque et al. 2016). An alternative to classical methods (passive samplers, mobile monitors) (Abad et al. 2022) is the use of indicator organisms (biomonitors), which prove to be sensitive species to absorbed PAHs (Sulistiyorini et al. 2022). Mosses are quite commonly used in biomonitoring (Carrieri et al. 2021; Jovan et al. 2021). They are used both as passive and active PAH samplers in open areas or in confined spaces (Vuković et al. 2014; Jayalath et al. 2020). The “moss-bag” technique is a method of active biomonitoring which consists of material exposed in mesh for a specified time (Ares et al. 2014). An essential element of this method is exposure time (Ares et al. 2012), so the assessment of the pollution of a given site/area should be closely related to this parameter and the determination of the effect of exposure time on emissions from various sources (Capozzi et al. 2021). Until now, biomonitoring studies on PAHs using mosses have typically involved short exposure periods: 4–12 weeks (De Nicola et al. 2013; Vingiani et al. 2015). These studies mainly concern the influence of the season and the vitality of the material during exposure (Capozzi et al. 2020; Gómez-Arroyo et al. 2020) or the comparison of the accumulation properties of various biomonitors (Capozzi et al. 2017, 2021). Conducting biomonitoring studies with longer exposure times is rare, and they are mainly concerned with heavy metal pollution (Saitanis et al. 2013; Milićević et al. 2017). In fact, only one example of “moss-bag” exposure over a 1-year period for monitoring changes in PAHs can be found in the literature (Foan et al. 2015). We found it appropriate and necessary to develop the literature research on the topic of using mosses to identify sources of PAHs. Firstly, we conducted a standard-period study using three moss species to monitor changes in PAH concentrations (Świsłowski et al. 2021a). But based on our own experience in the long-term application of mosses as heavy metal pollution biomonitors (Świsłowski et al. 2021c), we considered an important task to conduct research on PAH pollution monitoring in an urban area for 1 year.

The objectives of our study were (I) to determine the variability of concentrations of selected PAHs during annual exposure of mosses and identify sources of PAH emissions and (II) to show the differences between species during long-term active biomonitoring of these compounds.

## Material and methods

### Material

We used *Pleurozium schreberi* (Willd. ex Brid.) Mitt., (Ps), *Sphagnum fallax* (H. Klinggr.) H. Klinggr (Sf), and *Dicranum polysetum* Brid. (Dp) mosses for this study. We collected them in September 2020 in forests in the Swietokrzyskie Province in southeastern Poland (51° 7′ 19.61″ N; 20° 29′ 52.78″ E). The mosses were collected in accordance with national regulations (The Minister of Environment of the Republic of Poland 2014). Mosses have already been collected from these sites for other biomonitoring studies on PAH determination (Gałuszka 2007; Dołęgowska et al. 2021). Another criterion for selecting these species was their previous testing in another area (Świsłowski et al. 2021a). Moss samples were collected away from tree canopy cover, roads, or any anthropogenic activity in accordance with the guidelines of the ICP Vegetation protocol (ICP Vegetation 2020). Pretreatment (washing) before exposure was in line with our own previous study (Świsłowski et al. 2021b). The green parts only of the gametophytes were selected for chemical analysis (Boquete et al. 2014; Lazo et al. 2022).

### Methods

**Fig. 1:**
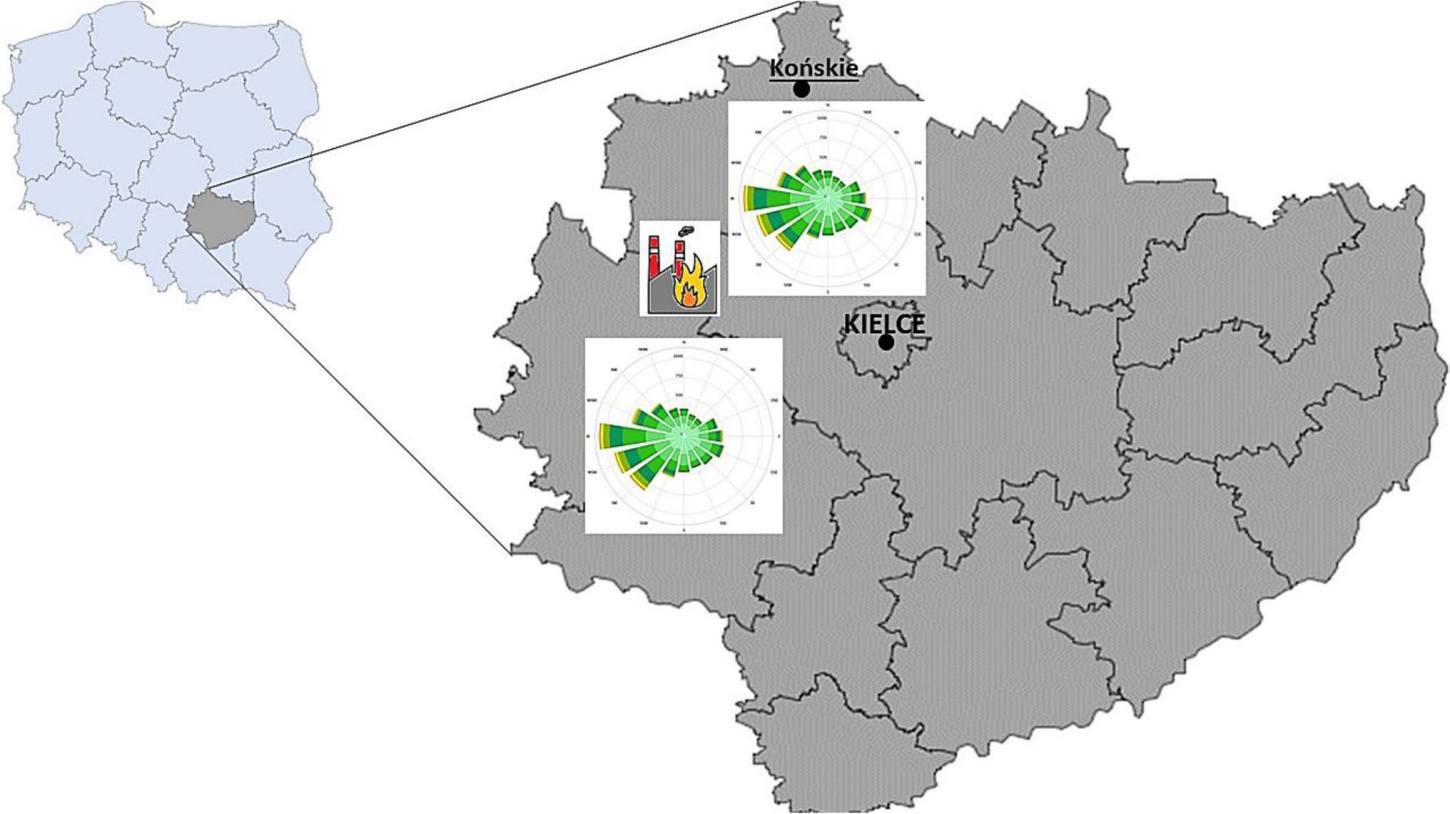
Research site. The map shows the Świętokrzyskie Voivodeship with the capital city of Kielce. The research location is also marked on the map in the upper part—Końskie City (51° 10′ 53″ N, 20° 25′ 26″ E). In addition, the village of Krasocin, which is approximately 50 km away from the research site, was marked with the symbol of a burning factory (more information in the text on this subject). For both places, the wind direction is shown, which blows from southwest (SW) to northeast (NE) (Meteoblue 2023)

Five grams of each moss species were packed into nylon meshes and displayed at an altitude of 1.50–2.00 m from the ground for a period of 1 year: from September 26, 2020, to September 25, 2021, a photo of the moss bags is shown in the Supplementary Material (Figures: S1 and S2). The samples were exposed to detect different types of contamination, for example, heating, winter season in Poland (October 2020–April 2021), and the non-heating periods (May–September 2021). The mosses were located exposed in Końskie City (51° 10′ 53″ N; 20° 25′ 26″ E). The study was carried out in an open space—samples were exposed 100 m away from any buildings and the nearest street roads (see Figure S1 in Supplementary Materials). Urbanistically, this city is very similar to other cities of its kind (ca. 18,000 inhabitants) in this area. The main industrial activity in the town is connected with involving foundry, construction, and transport (Przybytniowski and Dziekański 2020). The latest report of the Regional Department of Environmental Monitoring in Kielce (capital city of the province) showed that the level of benzo(a)pyrene (BaP) in particulate matter 10 µm (PM_10_) suspended dust in Końskie City exceeds the permissible standards (annual average concentration > 1 ng/m^3^). There is, therefore, a need to monitor and control this. The main source of exceedances in this report indicates the influence of emissions related to individual heating of buildings (Chief Inspectorate of Environmental Protection 2023). A total of 144 samples (24 physical “moss-bag” samples of each species × 2 bags per month × 3 replicates per sample) were analyzed. Control samples, which were also collected but not exposed (these are samples that have been exclusively collected from the forest and not bagged), were laid separately. Two bags of each moss species were removed after the 25th day of each month. *S. fallax* samples for the last 2 months were lost during the exposure period (probably due to strong wind gusts). The study site is shown in Fig. 1.

**Fig. 2:**
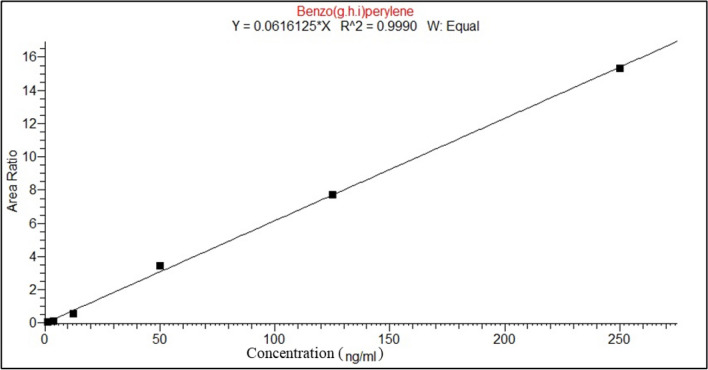
The linear calibration range for each analyte was from 1.25 to 250 ng·ml^−1^. Example of calibration for benzo(g,h,i)perylene

After exposure, 1 g of each sample was extracted by methanol (10 mL, HPLC grade) with the use of a laboratory shaker (GFL 3006). The extraction process lasts for 24 h. The obtained eluate was measured on a gas chromatograph (GC; Thermo Trace 1310) coupled with a triple quadrupole mass spectrometry detector (Thermo TSQTM 8000 Evo), a programmed temperature vaporizing injector (PTV) and an autosampler CTC Analytics AG, PAL LHX-xt. An analytical column (SCION 5 MS; length 30 m, diameter 0.25 mm, and film thickness of 0.25 µm) was used. The temperature program of the GC oven started off at 70 °C, progressed, at first, by 15 °C/min to 200 °C, and was kept for 5 min. Further, a temperature gradient of 8 °C/min was applied to a final temperature of 320 °C that was held for 5 min. A He gas flow of 2 mL/min was applied. All PAHs were determined using calibration with internal standards. Following PAHs were determined: naphthalene (NP), acenaphthylene (ACY), acenaphthene (ACE), fluorene (F), phenanthrene (Pn), anthracene (An), fluoranthene (Fl), pyrene (Py), chrysene (Ch), benzo(a)anthracene (BaA), benzo(b)fluoranthene (BbF), benzo(k)fluoranthene (BkF), benzo(a)pyrene (BaP), indeno(1.2.3)-cd_pyrene (IP), dibenzo(a.h)anthracene (Dah), and benzo(g.h.i)perylene (Bghi). The short names of the compounds are shown according to these authors (Tobiszewski and Namieśnik 2012; Ihunwo et al. 2019). The procedure for the determination of PAHs in mosses was identical to that adopted in a previous study (Świsłowski et al. 2021a), and the choice of PAHs determined relates to compounds that were analyzed in mosses by other authors (Concha-Graña et al. 2015; Jin et al. 2020). Four deuterated standards, chrysene d12, phenanthrene d10, naphthalene d8 and perylene d12, and mixed-standard Dr. Ehrenstorfer PAH-Mix 9, were used for the determination of PAH. The quantification limits (LoQ) were 1 µg·mL^−1^ for each analyte. As for the analytical methods, the MS parameter description for individual PAHs is provided in Table 1, and calibrations of MS are provided in Fig. 2. On the other hand, Figure S3 shows an example of a chromatogram of an analyzed moss sample, which is included in Supplementary Materials.

**Table 1:** MS parameters of PAH determination method

Analytes	Retention time (min)	Parent ion (m/z)	Production (m/z)	Collision energy (eV)	ISTD	LoD (ng·g^−1^)	LoQ (ng·g^−1^)
Naphthalene	4.45	128	102	20	1	0.29	0.96
Acenaphthylene	7.02	152	126	20	1	0.26	0.88
Acenaphthene	7.29	153	151	40	1	0.29	0.96
Fluorene	8.11	165	163	30	2	0.38	1.28
Phenanthrene	9.82	178	152	25	2	0.58	1.92
Anthracene	9.93	178	176	20	2	0.62	2.08
Fluoranthene	13.96	202	200	30	2	0.67	2.24
Pyrene	14.98	202	200	30	3	0.55	1.84
Chrysene	19.87	202	200	35	3	0.50	1.68
Benz(*a*)anthracene	19.98	228	226	30	3	0.62	2.08
Benzo(*b)*fluoranthene	23.27	252	250	30	4	0.58	1.92
Benzo(k)fluoranthene	23.35	252	250	30	4	0.65	2.16
Benzo[*a*]pyrene	24.14	252	250	30	4	0.53	1.76
Indeno(*1.2.3-cd*)pyrene	26.86	276	274	40	4	0.41	1.36
Dibenzo(*a.h*)anthracene	26.95	278	276	30	4	0.46	1.52
Benzo[*ghi*]perylene	27.41	276	274	30	4	0.30	0.99
Internal standards							
ISTD Naphthalene D8	4.45	136	108	25	1		
ISTD Phenanthrene D10	9.81	188	160	30	2		
ISTD Chrysene D12	19.90	240	236	30	3		
ISTD Perylene D12	24.31	264	260	30	4

Principal component analysis (PCA) was performed to explore associations between the measured PAHs and meteorological conditions. For this verification, Statistica software was used. The PCA is one of the basic multi-elemental analyses used in the evaluation of atmospheric deposition using biomonitoring (Krakovská et al. 2020). Statistical differences of selected PAHs were determined by the Pearson test. All statistical inferences were conducted at a *p* < 0.05 level.

## Results and discussion

**Fig. 3:**
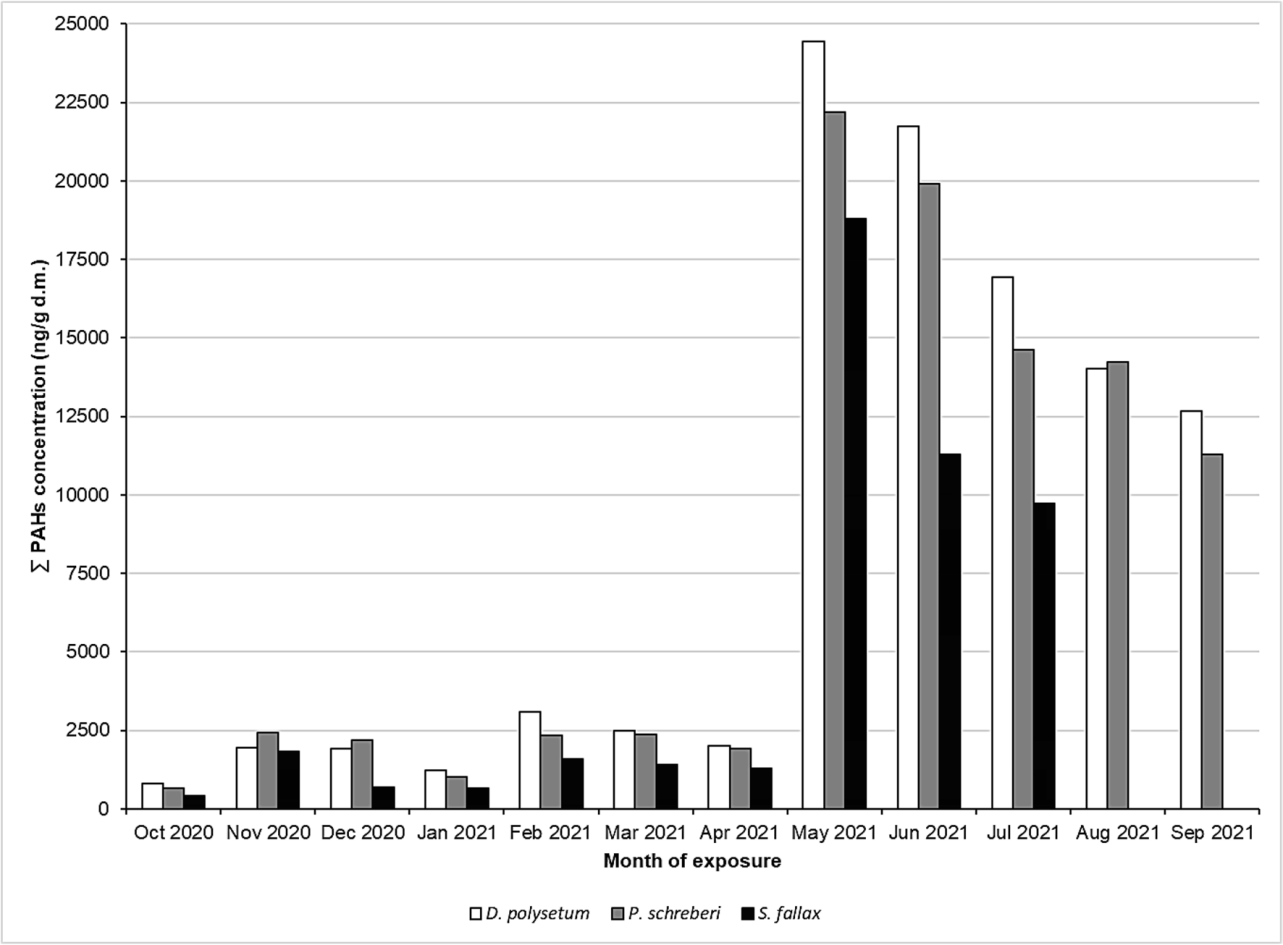
Seasonal changes in annual biomonitoring of the total concentrations of the 13 PAHs in the mosses (ng/g dry mass). Values indicate relative concentrations, so *C*_1_–*C*_0_, which is the post-exposure concentration (*C*_1_) of a given month minus the concentration of the unexposed control sample (*C*_0_). *S. fallax* samples for the last 2 months were lost during exposure (in accordance with the description contained in the methodology)

Naphthalene, acenaphthylene, and acenaphthene were not included in the 16 PAHs analyzed in the study, because their concentrations were below the detection/quantitation limit. Figure 3 shows the seasonal changes of the remaining 13 PAHs to their total concentration determined in the three moss species.

The results in Fig. 3 indicate the variability of PAH concentrations in mosses during the year. PAH concentrations vary practically every month in each species (see Table 2). A massive increase in concentrations was observed after the 8th month of exposure, which will be discussed later concerning the event that influenced this. During the annual exposure of the three moss species, three seasonal decreasing trends of changing total PAH concentrations can be observed: from November to January, from February to April, and from May to September. Generally, higher concentrations of air pollutants in winter than in summer are related to the heating season (Vuković et al. 2015). An in-house study comparing PAH concentrations in three moss species for the corresponding winter period indicates that Opole City, PL was more polluted than this study city—Końskie (Świsłowski et al. 2021a). The provincial city center was almost twice as polluted as the county town in the current study. Only the annual survey performed with active biomonitoring using *Hylocomium splendens* indicates how much the location of the survey affects the result. Mosses exposed near the Bértiz Nature Reserve (Bertiz Lordship Natural Park) in the 5th month reached a value of about 1100 ng/g dry weight PAH concentrations (Foan et al. 2015), whereas, for our mosses in the city, such values were already reached after the second month of exposure. This shows how much the place of exposure affects the result obtained (Sucharová and Holá 2014). Higher concentrations in the spring and summer period (second seasonal trend) may be related to the fact that with the increase in temperature, more dissolved PAHs volatilized into the atmosphere (Zhang et al. 2021). According to the previously mentioned report (Chief Inspectorate of Environmental Protection 2023), higher concentrations of lead, nickel, and cadmium in PM10 dust as well as PM10 dust itself were also observed in the spring and summer months (May–June) for the nearest monitoring station (50 km east of Końskie City). The third trend will be discussed later in the article. Table 2 shows a comparison of the total PAH concentrations accumulated by individual moss species.

**Table 2:** Comparison of differences in the accumulation of total PAH concentrations

Month	*D. polysetum* (Dp) (ng/g d.m.)	*P. schreberi* (Ps) (ng/g d.m.)	Difference from Dp (%)	*S. fallax* (Sf) (ng/g d.m.)	Difference from Dp (%)
Oct 2020	802	670	− 16.4	405	− 49.4
Nov 2020	1948	2437	+ 25.1	1840	− 5.51
Dec 2020	1919	2184	+ 13.8	694	− 63.8
Jan 2021	1234	1032	− 16.4	656	− 46.8
Feb 2021	3101	2341	− 24.5	1594	− 48.6
Mar 2021	2502	2373	− 5.15	1421	− 43.2
Apr 2021	2020	1927	− 4.60	1276	− 36.8
May 2021	24,451	22,182	− 9.28	18,782	− 23.2
Jun 2021	21,749	19,897	− 8.51	11,280	− 48.1
Jul 2021	16,928	14,611	− 13.7	9731	− 42.5
Aug 2021	14,029	14,217	+ 1.34	n.d	–
Sep 2021	12,666	11,275	− 11.0	n.d	–

*n.d.* no data (*S. fallax* samples for the last 2 months were lost during the exposure period in accordance with the description contained in the methodology chapter)

**Fig. 4:**
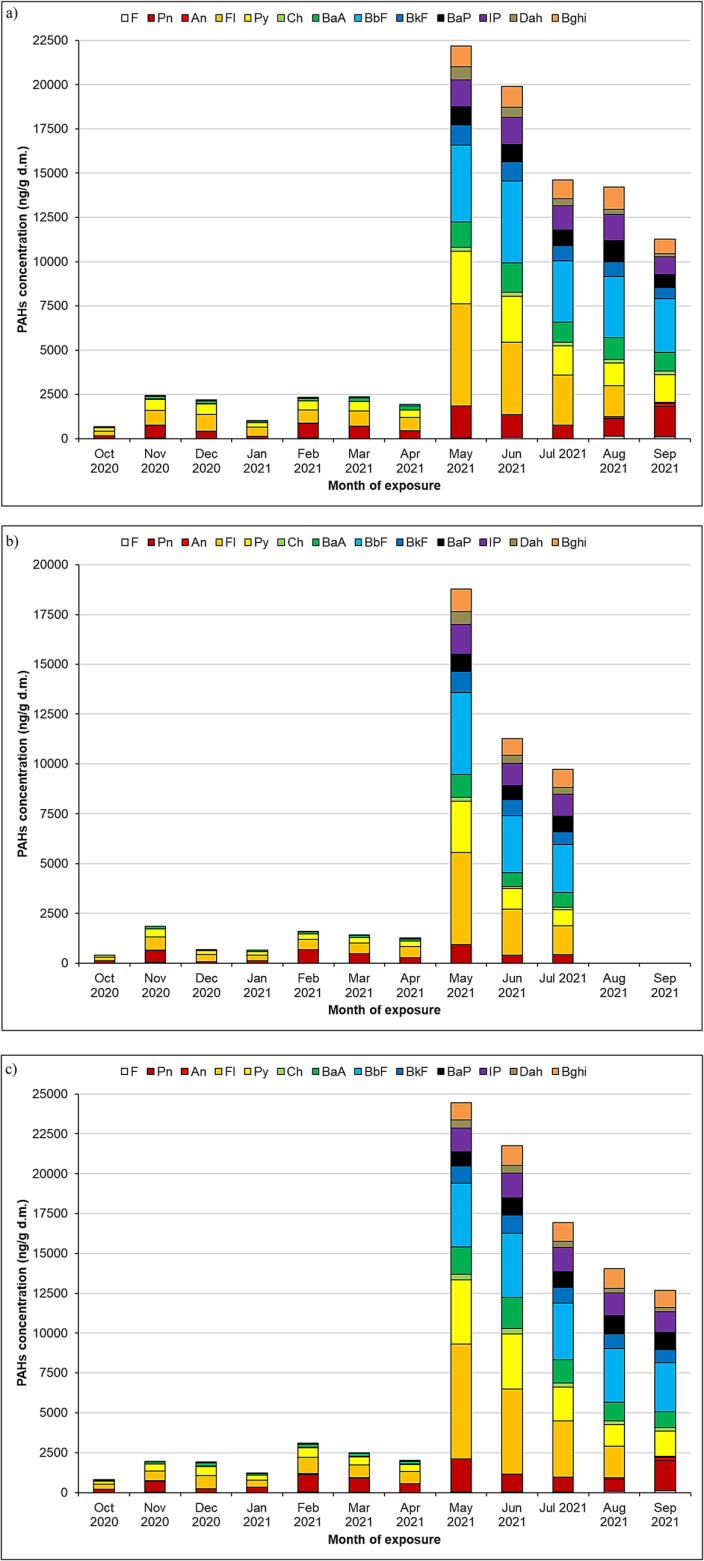
Seasonal changes in PAH concentrations (ng/g d.m.) in the **a** *Pleurozium schreberi*, **b** *Sphagnum fallax*, **c** and *Dicranum polysetum* mosses

Comparing the mosses from this study, they can be arranged in a series *D. polysetum* > *P. schreberi* > *S. fallax* in terms of decreasing concentrations of PAHs determined in their gametophytes (see also Fig. 3). In a previous experiment (Świsłowski et al. 2021a), the one that accumulates the most and therefore is the most useful for the work was *P. schreberi*, while in this work, it was *D. polysetum*, which accumulated on average 26.5% more than the other species (see Table 2). In another study, using passive biomonitoring in Kielce, *P. schreberi* accumulated ∑17 PAHs, a mean of 1371 mg/kg, compared to a mean of 1572 mg/kg in *H. splendens* (Dolegowska and Migaszewski 2011). For time series and success control studies, it is essential to compare the same seasonal period, due to the variation of the three moss species found in this study. This indicates differences in the PAH absorption capacity of the moss species, and therefore, it is necessary to use several species of mosses for the biomonitoring of these contaminants (Concha-Graña et al. 2015; Spagnuolo et al. 2017). The latest literature reviews do not report total concentrations of PAHs, and the species *D. polysetum* has not been used in active biomonitoring of PAHs so far (Mukhopadhyay et al. 2020; Gao et al. 2023). The structure of individual PAHs in the three moss species in the annual survey is shown in Fig. 4.

The results in Fig. 4 indicate three types of compositions of PAH compounds present in the mosses. Solid compounds that appeared in all months of exposure, regardless of species, viz., were as follows: phenanthrene, fluoranthene, pyrene, benzo(a)anthracene, and benzo(b)fluoranthene. Periodic compounds that occurred selectively, in selected months, with variable frequency were as follows: fluorene, anthracene, and chrysene. The last group of compounds, in turn, was observed as a significant change (also in the concentrations of the other PAHs) whose concentrations had not previously been determined in previous months, namely, benzo(k)fluoranthene, benzo(a)pyrene, indeno(1.2.3)-cd_pyrene, dibenzo(a.h)anthracene, and benzo(g.h.i)perylene. These significant changes in the concentration values of PAHs in all species of mosses indicate the appearance of an additional source of emissions, causing them to increase after 8 months of exposure (May 2021) compared to previously exposed samples (see Fig. 4). This change was influenced by an event that occurred on May 16, 2021, in Krasocin, 50 km southwest of the study site. The production halls of a toilet paper plant burned down on that day. To date, there have been no reports about this event, in the context of also scientific research, and the fire itself has only been reported in the local media (Zemsta and Barwinek 2021). Moss transplants *H. cupressiforme* reflected PAH depositions from recent pyrogenic events in a land historically devoted to agriculture, where recurrent waste burnings randomly occur (Capozzi et al. 2017). In another publication, a joint presence of benzo(a)pyrene and indeno(1,2,3,-cd)pyrene comes from the pyrolysis of scrap tires (Chen et al. 2007). As mentioned earlier, this is the impact of the carryover of pollutants caused by the factory fire. As a result, an additional five PAHs were determined in the mosses: benzo(k)fluoranthene (BkF), benzo(a)pyrene (BaP), indeno(1.2.3)-cd_pyrene (IP), dibenzo(a.h)anthracene (Dah), and benzo(g.h.i)perylene (Bghi). We can also find cases in the literature where post-wildfire PAH concentrations were significantly higher two to three times at two bogs (Zhang et al. 2022) or the impact of increasing PAH steepness. Historic Σ13PAH peak (~ 90 ng/g) is accompanied by a high concentration of retene in the *Sphagnum* peat cores, indicating that this peak probably represents the signature of a past fire event (Zhang et al. 2016a). Based on conservative ratios of Fl/(Fl + Py) and IP/(IP + Bghi), grass, wood, and coal combustion should be assigned as the source because, during the entire research period for these mosses, these ratios were 0.59–0.65 and 0.54–0.58, respectively (Tobiszewski and Namieśnik 2012). The common average proportion of new compounds in the structure of all PAHs for this period (May–September 2021) is 29.4%. So 1/3 of all compounds labeled in the mosses come from the influx of a source such as fire and the transfer of pollutants further away. In the following months (from the “fire” month onward), one can already see a decrease in pollutant concentrations, which is also noted in the literature that immediately after the fire, there is an increase in concentrations of PAHs, and concentrations of organic contaminants in mosses decreased a few months after the fire (Colabuono et al. 2015). A similar effect was also noted for the levels of PAHs in the pine bark, litter, and soil samples that became significantly elevated by the forest fire, but, after a few months, there were apparent decreasing trends in PAH levels (Choi 2014).

**Fig. 5:**
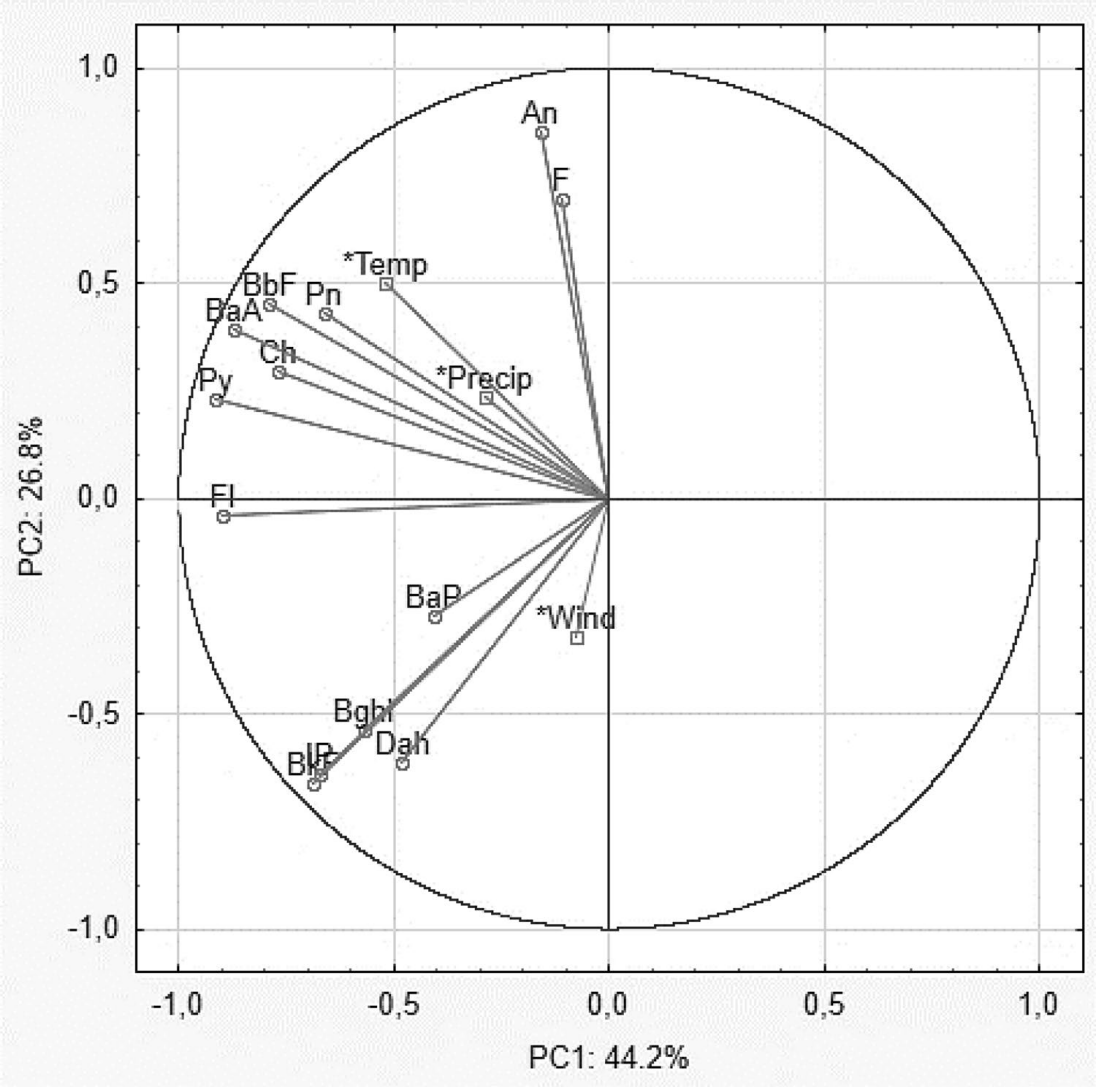
Principal component analysis illustrating the relationship between PAH concentrations and meteorological conditions. Fl fluoranthene, Py pyrene, Ch chrysene, BaA benzo(a)anthracene, BbF benzo(b)fluoranthene, BkF benzo(k)fluoranthene, BaP benzo(a)pyrene, IP indeno(1.2.3)-cd_pyrene, Dah dibenzo(a.h)anthracene, Bghi benzo(g.h.i)perylene, *temp temperature, *Precip precipitation

However, it is essential to consider also meteorological conditions that may contribute to this effect, so Fig. 5 shows a PCA diagram related to this.

Comparing meteorological data (wind direction), it should be concluded that the fire affected PAH concentrations in mosses. The wind in Krasocin and Końskie blows from southwest (SW) to northeast (NE), see Fig. 1 (Meteoblue 2023). As mentioned earlier, wind influenced the concentrations of five new PAHs, which were determined in mosses exposed for 8 months, after the plant fire. In contrast, the remaining PAHs are correlated with other environmental parameters: temperature and total precipitation in a given month (Fig. 5). Variables with an angle of 0° between their guides are completely positively correlated (Krakovská et al. 2020). The group of PAHs, Py, Ch, BaA, BbF, and Pn, has a strong correlation with each other (*r* was from 0.627 to 0.967 statistically significant, *p* < 0.05, see Table S1 in Supplementary Materials). This analysis confirms that meteorological variations play an essential role in pollutant accumulation (Choi 2014; Al-Alam et al. 2017) and take into account weather conditions (Dron et al. 2021; Jovan et al. 2021). PAH pollution in vegetation was produced by their deposition from combustion smoke (company fire). However, the low level of bioaccumulation depended on the limited exposure of the plants to smoke thanks to the smooth winds blowing in one literature example (Rey-Salgueiro et al. 2008). Otherwise, source apportionment indicates that biomass combustion from the neighboring countries contributed about 35–72% of PAHs in the air affected by different wind directions (Zhang et al. 2021). It has also been proven that distant emissions from the smelter may have an impact on moss contamination many kilometers away from the emission source (Cowden and Aherne 2019).

In the context of the analysis of the results and their discussion, it is also essential to emphasize the influence of moss biology on their accumulation of analytes (Świsłowski et al. 2021d). Many studies postulate the use of devitalized material, which is an effective monitor of contamination and has many advantages, according to the literature (Carrieri et al. 2021; Vázquez-Arias et al. 2024). However, it is essential to remember that the bioindicator is a living organism, not only a chemical sorbent (Boquete et al. 2017). The aspect of moss vitality and its effect on contaminant sorption should, therefore, be included in the research (Chen et al. 2015; Zhang et al. 2016b). However, as shown in another study, mosses rapidly enter a state of cryptobiosis during exposure, where PAH uptake and accumulation is a combination of adsorption and absorption (as passive mechanisms) where vitality plays a marginal role (Capozzi et al. 2020). Therefore, we have dispensed with such analyses (such as fluorescence and chlorophyll content testing) in this study. In our other work on biomonitoring with mosses carried out throughout, e.g., 3 months, we present the results of studies on, e.g., the vitality of mosses during their exposure (Świsłowski et al. 2021e, 2022).

## Conclusions

For the first time in an urbanized area, annual active biomonitoring was carried out using three moss species to assess atmospheric aerosol pollution by selected PAHs. The study showed seasonal changes in the concentrations of these compounds, which depended on the heating season, meteorological conditions, and the exposed moss species. A fire at a manufacturing plant resulted in an increase of an additional 5 PAHs in mosses after 8 months of their exposure. This demonstrates the feasibility of using the active biomonitoring method to assess and pinpoint the source of PAH air pollution. The species *D. polysetum* should be studied more in further studies using the “moss-bag” technique. The long-term biomonitoring study also indicated the need to use this biological method of air pollution assessment as a valuable tool in air status/quality monitoring. In Poland, PAH concentrations in atmospheric aerosol are tested selectively and only at selected measurement points. From the point of view of health protection, only benzene and benzo(a)pyrene are assessed. The use of mosses to assess the level of air pollution with these compounds allows for a wide range of research, including in rural areas, and thus, we can identify sources of pollution and protect people from the effects of PAHs in the air. In the future, more focus should also be placed on considering the biological aspect of moss life during biomonitoring studies of PAH air pollution and including them in the analyses of the concentrations obtained.

### Supplementary Information

Below is the link to the electronic supplementary material.

Supplementary file1 (DOCX 2353 KB)

## Data Availability

All data generated and analyzed during this study are available from the corresponding author on reasonable request.
