# Supplementary material for: One year of active moss biomonitoring in the identification of PAHs in an urbanized area—prospects and implications
Source: Environ Sci Pollut Res Int. 2024 May 28;31(26):38416–27. doi: 10.1007/s11356-024-33831-8 (PMC11189310; doi:10.1007/s11356-024-33831-8)
Supplement: Supplementary file 1 — Supplementary file1 (DOCX 2353 KB) [file 11356_2024_33831_MOESM1_ESM.docx]

*Supplementary Materials*

Paweł Świsłowski ^a,^*, Stanisław Wacławek ^b^, Vojtěch Antos ^b^, Inga Zinicovscaia ^c, d^ Małgorzata Rajfur ^a^, Maria Wacławek ^e^

**One year of active moss biomonitoring in the identification of PAHs in an urbanized area – Prospects and implications**

*^a^ Institute of Biology, University of Opole, Kominka 6,6a, 45-032 Opole, Poland*

*^b^ Institute for Nanomaterials, Advanced Technologies and Innovation, Technical University of Liberec, Studentská 1402/2, 461 17 Liberec 1, Czech Republic*

*^c^ Horia Hulubei National Institute for R&D in Physics and Nuclear Engineering, Bucharest Magurele, 30 Reactorului Str. MG-6, Bucharest, Romania*

*^d^ The Institute of Chemistry, Moldova State University, 3 Academiei Str., 2028 Chisinau, Moldova*

*^e^ Society of Ecological Chemistry and Engineering, Zawiszaków 3/103, 45-288 Opole, Poland*

* Corresponding author: pawel.swislowski@uni.opole.pl


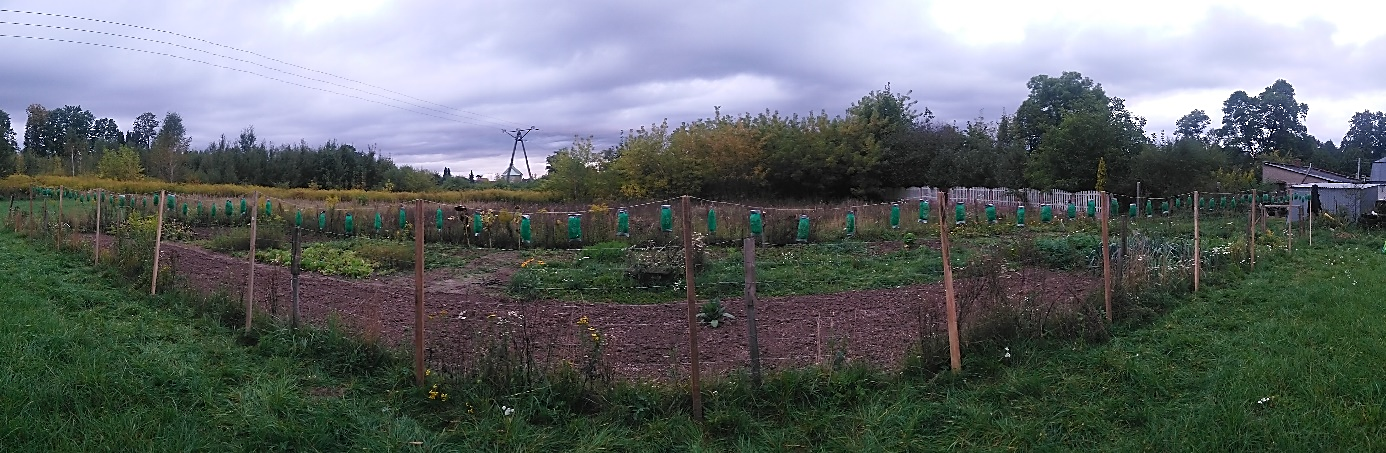


Figure S1. Photo of ‘moss-bags’ in the study site (photo by P. Świsłowski)


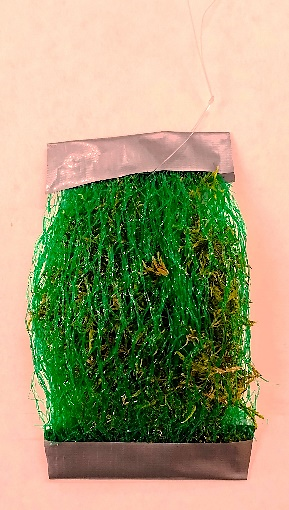


Figure S2. Photo of ‘moss-bag’ before exposition with *P. schreberi* moss (photo by P. Świsłowski)


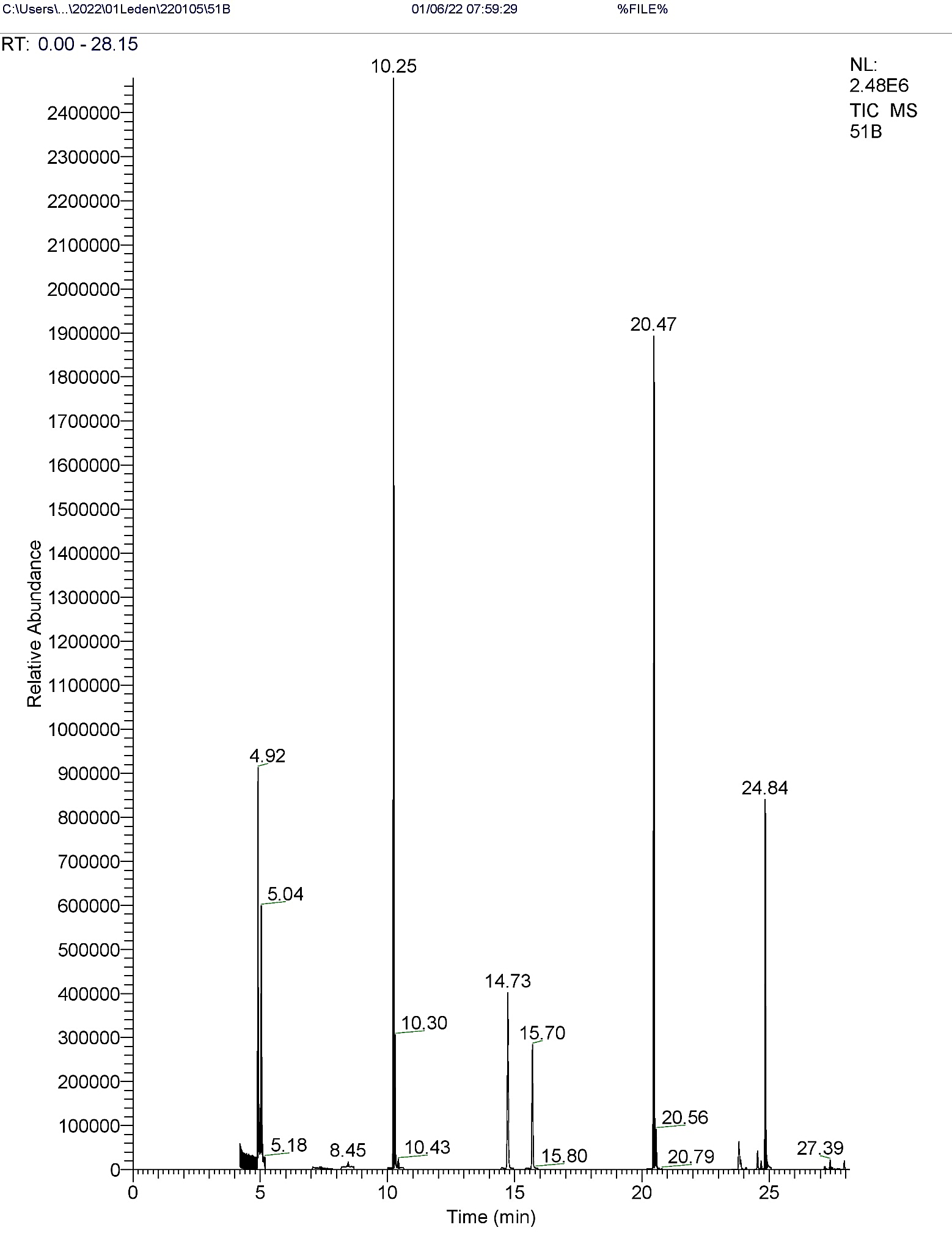


Figure S3. One example of a chromatogram of an analyzed moss sample (51B1- number of sample)

**Table S1**. Pearson coefficients for selected PAHs

|  | **Mean** | ***SD*** | **Pn** | **Py** | **Ch** | **BaA** | **BbF** |
| --- | --- | --- | --- | --- | --- | --- | --- |
| **Pn** | 728 | 528 | 1.000 | 0.783 | 0.627 | 0.721 | 0.661 |
| **Py** | 1029 | 1034 | 0.783 | 1.000 | 0.886 | 0.938 | 0.878 |
| **Ch** | 161 | 110 | 0.627 | 0.886 | 1.000 | 0.960 | 0.888 |
| **BaA** | 570 | 605 | 0.721 | 0.938 | 0.960 | 1.000 | 0.967 |
| **BbF** | 1627 | 1829 | 0.661 | 0.878 | 0.888 | 0.967 | 1.000 |

correlation coefficients marked in red are significant with *p* < 0.05
